# The journey of aftercare for Australia’s First Nations families whose child had sustained a burn injury: a qualitative study

**DOI:** 10.1186/s12913-020-05404-1

**Published:** 2020-06-13

**Authors:** Julieann Coombes, Kate Hunter, Tamara Mackean, Rebecca Ivers

**Affiliations:** 1 The George Institute for Global Health, Level 5, 1 King St, Newtown, NSW 2042 Australia; 2 University of New South Wales, Room 325A, Samuels Building, Sydney, 2052 Australia

**Keywords:** First nations, Children, Barriers, Facilitators, Burns, Aftercare, Health workers, Liaison officers

## Abstract

**Background:**

Access to ongoing multidisciplinary healthcare services for children who have sustained a burn injury is critical for optimal recovery. This paper reports on barriers and facilitators to culturally safe and appropriate burn aftercare for Australia’s First Nations children. The voices of First Nations families whose child had sustained a serious burn are central to this paper.

**Methods:**

Eighteen families, which consisted of 59 family members, of children younger than 16 years who had sustained a burn injury were asked to describe their own journey in accessing appropriate burn aftercare. Interviews were conducted in the families’ homes using yarning (dialogue) and Dadirri (deep listening) as Indigenous research methods. Data was gathered in South Australia, the Northern Territory, Queensland and New South Wales, Australia. Using a cyclic process, transcripts and emerging themes were sent back to participants, and a collaborative approach was used to conduct the final analysis.

**Results:**

Lack of culturally safe communication between service providers and family members, in addition to institutionalised racism, were found to be the major barriers to accessing healthcare services. Distance to medical treatment also impacted children’s access to aftercare. Involvement of First Nations Health Workers and/or Liaison Officers working with health providers, the child and family members, was found to be an important facilitator in reducing miscommunication and alleviating fear and anxiety in the children and families.

**Conclusion:**

There are significant barriers to access to aftercare following a serious burn including miscommunication, lack of cultural safety, distance to medical treatment and racism. However, these can be largely mitigated when First Nations families have input into the care received and the care needed for ongoing burn care to be effective and are supported by First Nations Health/Liaison Officers support.

## Background

Burn injuries can be devastating not only for the injured child but also for their family and their community. Australia’s First Nations children experience burns at a rate at least double that of non-Indigenous children [1]. To date, there has been limited published research on burns in Australia’s First Nations children, and what exists focuses largely on describing the burden using routinely collected data [2–4]. More recent work led by our team has highlighted the need for changes in policies and practices within the area of burns [5, 6]. The accessibility of high-quality aftercare for First Nations children, who constitute a high proportion of burns patients, predominantly those from regional and remote settings, is particularly important, given the complexity of long-term burns care, where access to appropriate treatment is essential to produce good long-term outcomes [7]. It is well-documented that burns can cause physical discomfort, lifelong scarring, and overwhelming psychological distress. Burn injuries also have an impact on the injured person’s family including dislocation from other family members and their communities [8]. Confusion about and a lack of understanding of healthcare procedures and protocols reinforce anxiety and mistrust of healthcare providers [9]. Ensuring that treatment and ongoing care is planned such that families are readily able to access appropriate care is an essential aspect of long-term burns care, but it is unclear how well this occurs in the treatment of burns in First Nations children. There are documented gaps in access to healthcare by First Nations people, both in primary and tertiary care settings [10], and there are studies highlighting delays which is suggestive of problems in the interface between primary care and the acute care sectors [11]. However, there are no studies on how First Nations children who have sustained a significant burn injury access burn aftercare once they have left the hospital burns unit. This study will focus on this gap by listening to the voices of children and their families through yarning (dialogue) and Dadirri (deep listening) about their experiences and the barriers or facilitators they experienced in accessing appropriate burn aftercare [12, 13].

## Methods

Indigenous methods were applied to this research from inception through to data analysis through the standpoint of a First Nations researcher [14]. The use of Indigenous methods prioritises the voices of the families whose child had sustained a burn injury and needed to access burn aftercare [15]. Indigenous methods employed in this research include yarning and Dadirri, both recognised as techniques to gather stories of families’ lived experiences in partnership with the researcher [16, 17]. Yarning and Dadirri are forms of communication that are culturally safe and culturally appropriate ways of engaging in conversation [12, 13, 18]. The primary researcher is an Australian First Nations woman whose aim was to decolonise this research project by privileging the voices of the families [17].

### Data collection

Australia’s First Nation families of 18 children younger than 16 years who had sustained a burn injury were asked to describe their lived experiences of their own journey from the time of injury to the time of recovery including accessing appropriate burn aftercare. Families were recruited purposively from a larger national study examining burn care in Australia’s First Nations Children (Ivers et al., 2015). Families were selected to ensure diversity of experience and access to burn aftercare. Data were gathered in South Australia, the Northern Territory, Queensland and New South Wales, Australia, from city, urban, remote and very remote areas. Families were diverse, from those having both parents to one parent homes where the carer was mother, father or a grandparent. Some families had a parent employed while others were unemployed. Two families from Northern Territory and one family from the Torres Strait Islands had English as their second language however no interpreter was needed for our yarns as their English was proficient and the families were comfortable communicating in English.

Families were invited to yarn about their child and family’s experiences between 2017 and 2018. All families had either one or both parents that identified as Aboriginal or Torres Strait Islander. Yarns were conducted in the family’s home and community using yarning and Dadirri as Indigenous research methods of storytelling. As yarning and Dadirri occurred within families’ home and community, there were no time constraints therefore sessions could last hours while sharing a meal, stories and connecting with one another. As experiences from the acute phase could impact on experiences of and access to burn aftercare it was important to understand the whole journey from the initial burn incident, the aftercare, through to the time of yarning together. Once the rapport and connection were established an audio recorder was brought out and, with the permission of the family, recording started.

### Analysis

The family’s stories were audio-recorded and transcribed verbatim. The professional transcription service is based in Victoria, Australia. Using a cyclic process JC first reviewed the transcripts using a grounded theory approach and emerging themes were established. Families were then sent the emerging themes and their own manuscripts by mail for review. Phone calls were made to each family to yarn about the findings in their transcripts, this collaborative approach taken with each family contributed to the final analysis.

### Ethics

Ethic approvals for the study were gained from seven Human Research Ethics Committees (HRECs) in Australia. The ethical procedure of this study referred to the relevant guidelines for Ethical Conduct in Aboriginal and Torres Strait Islander Health Research [19, 20].

## Results

In New South Wales we yarned with 5 families who were from urban areas, remote areas and very remote areas. In South Australia we yarned to 2 families that were from urban areas and 1 from a remote area. In Queensland, 2 families were from the city, 2 from an urban area, 2 were from a remote community, 1 family was from a very remote area and 1 family lived in the Torres Strait Islands. Two families from the Northern Territory, which included extended family members, had to relocate from a very remote community in an urban area due to ongoing aftercare for the burn injury. Three families had lost their employment due to their child’s burn injury and the time spent away from their homes and other family members. Eighteen children who sustained a burn injury and members of their families participate in the yarning and Dadirri this included mothers, fathers, Aunties, cousins, grandparents and siblings in total 59 family members participated in the yarning.

### Communication

Families expressed the need for better communication from health workers. They described feeling confused when they left the burns unit after experiencing a lack of information about the burn care and what was expected once they had left the burns unit. Medical jargon and not being told what would happen next to their child was disconcerting for families, leaving them in fear and feeling angry. Communication lacking sensitivity and person-centred care was confronting to families trying to calm their child for their aftercare treatment (Table 1).

**Table 1:** Communication needs

Lack of information causing confusion	“… I think there is no information or good communication. Because like, you just don’t get, like we tried, some things that you don’t understand what they’re saying, you not getting no information … Like, you know, like with his hand. And then with Madeline,^a^ I’m getting phone calls saying, this appointment from the clinic, to see the doctors, and then all of a sudden they postpone it, and then say, we’ve got the next one. But that’s not with the physio. So they do the physio and the doctor on the same day, and then they’re expecting us to go down there to see a physio but then the doctor keeps cancelling, I don’t know when the doctor’s going to see her, ‘cause they keep cancelling. It is sort of a mix up.” “I was kept in the dark, and it was just the bits that Brit^a^ was feeding back and that I was reading her notes to get some of the information, but then trying to ask questions, it’s like, got to wait until the doctor comes. Doctor comes at 9 o’clock in the morning, I’m not there until 10 o’clock because I just – I can’t get up there, by the time I get the other kids organised, so, I’ve missed everything, and the doctors wouldn’t come back and speak to you because they were busy.”
Gatekeeping of medical information	No-one would talk to us, no-one would tell us what was going on. It ended up going all night, where they went, “Oh, we don’t know what’s going on yet. We’ll leave it in the morning until the doctor comes in.” Finally, the doctor came in the next day... Yeah, and then he goes, “Oh yeah, yeah, we’ll get it sorted. We’ll have to probably send her to [the city] hospital. And we’ll get that organised.” Anyway, so it probably hit about lunch-time, nothing, heard nothing, like, what’s going on with the flights? What’s going on with everything? And they just kept going, “Oh, we don’t know, we don’t know, we don’t know.” And all this type of stuff, about two o’clock, I went, I can’t stand this anymore … This is ridiculous. They’re not telling me anything. They’re just fobbing me off all the time. So I ended up getting the contact numbers for the Burn Clinic [city hospital] and started contacting them myself. She [nurse from city burns unit] went, “We don’t have anything here for her. We don’t know what you’re talking about.” So then she got all the details over the phone and said, “We’ll admit her now. We’ll get onto the [country hospital] and we’ll get it sorted.” And within half an hour to an hour, it was sorted. The doctors come in and said, “Oh, we’ve just been in contact.” And I said, “No you didn’t. We just contacted them. The burn clinic has just rang you.” And they’ve all kind of went, “Oh”... But the country hospital were atrocious. Absolutely atrocious. I couldn’t believe it. They just left you there, you know. Yeah, it was just, like, horrible.
lack of patient/family centred care	“And screaming the way he is, you know, and I said, “Are you sure you’re doing it right?” I used to bath him. I said stop it and just wait and let him cool down and they said, “You need to leave, Miss”. Yeah, didn’t like that much. You know I wish they’d be a bit gentle with how they go because they’re not the ones who’re burnt. Or feeling all the pain, and yeah like, as parents we’ve got to watch, and watch our kids go through that pain. But yeah, no one doesn’t want to be - no parents want to be put outside the room to let them handle your kids.” “The doctor came in and said that they were going to take him into the room and pop the blisters and pull the skin off, but I don’t think the doctor realised a four-year-old understands that, so then he was absolutely, “No, it’s not happening, I’m not doing it,” We had to sort of say, “Now Bradie^a^, that’s not exactly what’s going to happen; you’re going to -” and so we had to explain it in a four-year-old way instead, so I think that they just don’t realise what age group they’re dealing with. Just being aware of the fact that that child could hear you. He can hear everything you’re saying. He’s not stupid, and he is four. So he knows, if someone’s going to pull his skin off, I can’t imagine what he would have thought after being burnt what they were going to do. I mean, the doctors probably could be more understanding of children – I know they’re run off their feet, but probably more educated on talking around the child, only because that was fairly traumatic for him when he sort of heard those small things that he could understand and he was just absolutely terrified and it escalated the situation when he was only just calming down.”
Medical jargon	“[He] just keep doing his physio and OT (Occupational Therapist) to make his body and his muscles stronger … He has to wear these suits here, his protection burn suits to make his burns like smoother. They [medical staff] talk all these words and that’s gone straight over my head, you don’t understand what they’re saying you don’t know what they are doing next**.**” “Oh, yes, sometimes I didn’t understand what they were talking about, them words were too big medical words. I didn’t have anyone with me and I tried to ask the nurse she kinda explained it to me about more dressings.”
Lack of person-centred care	I think the plastic surgeon that came down to have a look at him, he was on his phone talking to the other person [surgeon]; I thought that was a bit impersonal. I think if you’re going to do that, come in, check, go out and make the call then come back in. Don’t call when you’re standing in with the people; thought that was very rude. The surgeon could have come down and have a look and so you weren’t reassured, you were thinking, oh, he’s just sent his lackey down to have a look at you. Your kid who to you is the most important thing to you in the world you don’t know that there’s 100 other kids who are doing it, but they’re the most important thing to you.

^a^Not child’s real name

### Transport

Overall, transport was an issue that affected every family. Parking at hospitals was costly, sometimes resulting in fines due to appointment times being longer than expected. Distances from hospitals where aftercare was received caused stress on family budgets, schooling and work commitments. Appointments were made by the burns clinic and were not flexible in situations where families had other children to take into consideration (Table 2).

**Table 2:** Impact of travel

Burden and impact of travel and cost of parking fees for aftercare appointments	“The appointments taking longer than what you’re allocated in your time, and you can’t just leave her there and rush out and fix up the parking meter because you’ve got to park miles away, so, yeah, it was – we ended up with a few parking fines.” “I said just take them to the park because otherwise you pay a motza of parking here and I’m like, nah, just go around the street and take them to the park and I’ll call him when we need to be picked up, so yeah.” “And the petrol … Last time we travelled all the way down, and because there’s nowhere to stay we just travel all the way back home. Seven hours down and seven hours back for appointments. My two boys have been affected because there’s been a lot of times where I’ve also had to take them out of school. So my little boy has missed out on a fair bit of school because the only time that we would have to come down here for the dressings or for certain appointments that [the country hospital] don’t provide, I’ve had to take him because I can’t leave him there. So he’s missed out on a fair bit of school, Kath^a^ also. You know, she’s in high school now and I’ve got to drive down here, have appointments, then drive back. That’s a minimum of 3 days if you want to push it, of high school days every month or two. Yeah, it’s really affecting her school attendance as well as my little boy’s school attendance, which isn’t good.”
Intransigent appointment times	“And my disabled son, goes to school on his own schedule, if he wakes up at 8.30, 9 o’clock, then I can get him off to school, but I have to drop him off at school and I explain that to them and they still made her appointments for first thing in the morning when I ask them for later appointments, and it’s like, really? So, we were late, so then they’d make us wait, so we could be there anywhere from 4 hours, I think, was our longest appointment.”

^a^Not child’s real name

### Child, parent/carer, family support

One of the biggest challenge’s families highlighted was the lack of local support or services for aftercare treatment. Services, nurses or allied health workers trained in the area of burns aftercare was especially important. Written, culturally appropriate information that was easily understood about the aftercare needed with contact numbers was a request each family shared. The need for contact numbers for local support agencies to assist with emotional support for family, child or single parents was also commonly voiced (Table 3).

**Table 3:** Family support

Lack of local service providers disrupts family routines	“It would have been easier if maybe somebody could have organised someone to come here to change his bandages, instead of me taking all the kids down to the hospital all the time. They do it after you have a baby, they come out and check on you in your house, so I’m not sure why something wasn’t organised for them to come and change the bandages here, instead of making appointments every second day to come down [to the city hospital] with three or four other kids in tow. So yeah, it was pretty annoying. There are no local people to do the dressing here.” “We’re all very tired. And my second eldest has … and possible autism. So, it’s hard on him as well, going to other people’s houses. The kids have had to miss a bit of school. I miss things that they do at school, as well as taking them to school. So, Maison^a^, the second oldest, missed out on the pet parade and I organised for a friend to take him, Ellie the dog, and she (Ellie) didn’t feel like it. So, Maison^a^ had no pet for the pet parade, because I was in Sydney with Luca^a^. They get split up sometimes having to stay at different people’s places for days.”
Needed support for single parent families	“Mine would be the lack of support for single parents. When you have no family where you live and they want to fly you out, but you can’t get a flight out because you have another child and they won’t take extra children on the flight and then you have to drive with a kid that has an injury of a burn, you’ve got to drive nine hours to get there by a certain time and also the hospital that don’t cater for single parents to have to actually have the kids stay there because there might not be another option.”
The need for information for ongoing emotional support	“An information pack would be good to step you through all the emotions you go through and probably even the emotions that you’re going to feel after it happens and who to go to for help. You run through it constantly for days. You can’t sleep because you just – it’s always running in your head – up until you know that they’re going to be ok. Once I knew that it was all okay and that we’d finished the process, then it was – it sort of subsided, it went away, but in those times, you just constantly were thinking what could you have done, and you’d have dreams about what would have happened if you didn’t have the ice, the water, you went through all those emotions of what would have happened if we didn’t have that.”

^a^Not the child’s real name

### Family separation

Strong emotions were expressed when yarning about family disconnection. Families were separated for long periods of time and those with large families had to split up their other family members causing detachment. Being separated from their other children caused additional stress to the parent/carer. The child who had needed to travel large distances for aftercare became distressed at being separated from other siblings (Table 4).

**Table 4:** Family separation

Fractured family causing detachment from self, people, Country	“It is when you’ve got 13 children it’s very hard. And then like leaving them to go to [city burn clinic], when you think of taking them and all the way down there, it’s just not fair. It’s a long way away. If something happens you can’t say, “I’ll be there now,” or, you know? I worried being away from them.” “Yeah it has affected us a lot because it’s like split us all up, you know, with the – with the other two being away separate from us. I’ve only got these two and yeah, and it’s like he doesn’t really understand yet like what’s happened and that. He – like he’s yeah, doesn’t realise yet of the outcome of the burn and that and he doesn’t talk about it, you know, and when you ask him he says, “No it’s all right I don’t worry about it.” Yeah, but later on down the track I think he will when he gets a bigger boy, yeah, when he starts growing into a teenager side.” “I felt sad when I was down there because you don’t know, you know no-one. Too far away from families. I was lost down there. My boy was a bit sad too. Everyone here is related or ours. Because everyone’s cousins and all around here, and over the hill. I’ve got sisters up town there, and brother. This our community we help each other.”

### Racism

The families told of their experiences of racism they had endured while engaging in Australia’s healthcare systems. Families told of feeling judged and disempowered and described the colonialism they experienced as a family while their child was being treated for the burn injury (Table 5).

**Table 5:** Racism

Assumptions	“Well another thing, I mean we were in the room by ourselves and the nurse, … walks in and says, “You’re going to be right, your people’s come into the room.” And I went, “Huh?” “Oh, no you’ll be right, all your people are coming to the room.” It was a family, Indigenous family that got burnt out of their home and all that smoke inhalation. That was the whole family, at the same time that happens to you. Yeah. My people were coming. I didn’t know them from a bar of soap. They’re not our bloody people. Yeah. So ‘cause I’m Indigenous and that’s how people put them all in the one room.”
Judgementalism	“If my kids come and did this it would be a totally different outcome and we know it, we know it. We see it every day the difference, our kids are judged to the other kids. They think our kids are wild, they just playing”
Interpersonal racism	“One night there was a light switch that wasn’t working, that was broken, and I went to turn that light switch off but I couldn’t because it was zapping green behind the thing so I left it because I didn’t get zapped and then the nurse came in that night and I said to the nurse, “Excuse me but this light is broken you know we’re not going to lay down with the light on all night, I have to get up 6:30 in the morning and get Matty^a^ ready.” And she turned around and she said, “Well love you’ll just have to get a sheet or a towel and just put it over your eyes.” And I said, “Oh yeah, okay then thank you.” Next day this nurse walks in and she was talking real smart to me. She goes, “Oh the light’s working. Yeah you might have brains not to go and touch it, you might have got zapped you know.” They think we are dumb because we are Aboriginal.”
Racism in the form of punishment	“Mark^a^ didn’t want them doing anything to him because he thought that you know, they might hurt him and he was using it in a like swearing way towards them, and they just kind of thought no, we can’t deal with him. You know they all was like that, “No, we can’t deal with this kid,” you know, and like a couple times he was swearing really loud and they took the TV away from him to try and learn him. Took his food away and put it out on the table outside. Took his lunch, got his lunch took it outside and put it on the table. And took the cable from the TV, took it away. And I said “there was white kids in there doing that.” And they was, really noisy with that. White children was in there and they was doing the same and no, TV didn’t go off nothing, they had lunch, they [nurses] was like yeah, and rolled eyes at me.”
Lack of cultural safety	“And it’s when you’re a good mother and you feel like discriminated on that … Seeing people that look like junkies in there, and they get treated worse. Indigenous, because they look like junkies they get treated bad too. Another Indigenous family waiting, they were treating them really bad just because of the kids playing.” “They just set us the rules for us, oh yeah some are racist, because like some of them, they’re – oh they’re really, just not really nice. It makes you not want to come back.”
Disrespect leading to disempowerment	But they need to better respect the parents you know of the children that’s going through this because we’ve been through enough you know. We were stressed right out from you know, your kid nearly dying to helping your kid through the process of getting better and healing. You know, they just need to start respecting parents and you know, treat them kind. You know, and not being sour to people, smiling at them and asking them are you all right, you know, do you want help with that, you know, or something like that. They make us feel like we are dumb and can’t do nothing to help our child.” “They wanted to take Jack^a^ to [the city hospital] and I said, “I can’t drive.” So they said, “Well, he really needs to go to [the city hospital] but we’ll organise an ambulance to [our country hospital] for a dressing.” The nurse here said to me, “They don’t worry about the people in the scrub” [and] the person from [the city hospital] rang and said to me, “Well, move to the city and you’ll get treated better.”
Lack of professionalism contributing to ongoing systemic racism	When we walk in the room you know by the look on the face that they [nurses] think we are all the same, no good “… we try to change but we’re still the same to everyone … even the nurse they can just look at us and they [nurses] think he’s still the same … You can never prove yourself.” “They just set us the rules for us … some are racist, because like some of them, they’re look at us funny, oh they’re really just not really nice. It makes you not want to come back.”

^a^Not the child’s real name

### Healing in rural and remote communities

Staying in remote and rural communities with family and support systems was also identified as important for the ongoing health and wellbeing of the child and family. Families expressed the need for practical information on burn aftercare, as well as the aftercare being accessible to them in community clinics to lessen the stress and burden of travel and family dislocation (Table 6).

**Table 6:** Community support systems

Importance of family, home and Country in aftercare	“We did get help in the community. We did get a lot of support. Community helped a lot with my other kids, like family. Family helped rub cream on him all the time, to give me a break. There was always someone to take my daughter to her appointments.” “Yeah, there’s three – our three sisters and two brothers and all our kids from our siblings, all our nieces and nephews, they all call us mums and dads. There’s three mums and two dads plus grandfathers and other mums. But we’re a tight little group, but all Aboriginal families have all got that connection with kids. It would be good if we could get help from home hospital and stay together. No matter that’s your – my big sister, that’s your mum too, and so on and so on. You can have 10 mums; be the richest kid in the world.”
Information needed for the parent to help with home aftercare.	“I think just someone that I can actually talk to and just say hey, look, is this normal, is this not? would be good. And just, even if it’s phone contact once a month or something like that, just to touch base and say how’s things going, then, yeah. But it was – I’ve been through one child having open heart surgery but this was completely different. It’s, kind of, this threw me because I didn’t have any information I didn’t know what to do when we got home.”
Training needed for remote and rural health workers in burn aftercare	“Because even [the country hospital] when they sent us over to do it, the dressing, the lady was lovely, you know, can’t be helped, but Layla^a^ wasn’t happy with the dressing. She was scared it was going to get infected. You know, like I wish we had more people trained for the country. Country nurses need teaching with burn dressings.”

^a^Not the child’s real name

### Trauma

Trauma was evident when families in the study spoke about their child’s burn injury and the family’s journey through the healing processes. Although the child was treated for the burn injury in the burns unit, during the long process of aftercare there was no recognition or treatment for the psychological effects resulting from the injury for the child, or the one who caused the injury, or the family. A family experienced trauma through the removal of a sibling of the injured child to welfare other families were traumatised by fear of their child being removed (Table *7*).

**Table 7:** Trauma

Trauma cumulative and/or unrecognised	“See and then we’ve got to move around and do everything now, we’ve got to transfer and leave our home our family, he has to be close to [city hospital]. Yeah, I don’t want our family broken. It would be good to have just one person to help our family stay together. My kids are all over the place.” “Couldn’t sleep over it all. Just seeing them on fire like a matchstick, still after a year can’t sleep ‘cause it’s very stressful. I cried a lot by myself.”
Effects of the trauma on other family members	“My big sister Immy^a^, she said, “No, this boy’s really broken, Natasha^a^.” I said, “Yeah, poor darling.” His mother had to take him into hospital and the clinic there where they stay in the community and get him some sort of help, try and help him with a bit of sleeping pills, or something, put him to sleep, because he used to get up and just cry for Andrew^a^ and constantly trying to ring up and find out how Andrew^a^ was and kept saying sorry over and over on the phone, and that was sad for me.” "He [Andrew’s^a^ brother] sometimes he gets angry and he goes, “Yous are blaming me, yous are blaming me for Andrew^a^.” Yeah but he just hides his things and he doesn’t talk about it or let it out … Yeah and he was missing me as well when I was gone. Like he wasn’t understanding properly why mum and Andrew^a^ are gone for so long. Yeah and we tried telling him but we have to go because Andrew^a^ needs you know, proper care and proper treatment … Yeah, and then he [brother] thought that we gave him away that’s what he’s, that’s the other thing he’s thinking and we tell him, “We never gave you away. We’re just waiting till we get this house and then you’re coming back to us.”
Trauma of child removal by child services and the unnecessary reporting to child services	“There seems to be a lot of DoCS (Department of Community Services) calling on our kids. it’s like it was our fault and like they made us – made me, my partner he was there, we walked in the room with the DoCS and I just jumped up and I said, “What, I don’t care, you know, I’m sick of this, I’ve never done [swearing] nothing wrong myself.” Well, I just slammed the door and my partner said, “You shouldn’t have done that,” you know he was scared” "They didn’t (treat us well) Up – while we were down there the DoCS worker come because nurses, apparently the DoCs lady told me that they Chinese whisper in the hospitals. So little Chinese whispers caused DoCS onto us. So that was sort of very upsetting for me at the time as well, thinking what the hell did we do? It was a 19 year old that done it. That had all got fixed up, as we came home we – the police chucked it out the window as the DoCS people said, and the DoCS knew, like it was an accident … But yeah, that – you didn’t feel really safe at all. “The DoCS people … they were investigating Sam’s^a^ burn … They [hospital] must have had a report or something that he’s burned, but they were investigating how it happened. I think they’ve closed the case now. They rang up and said they were going to close it, but I haven’t heard anything from them at all.” “Dad was left home with our other kids and Derrick^a^ felt bad and he ran away and hid in the big bin and slept there, in the morning the big truck come to empty the bin and thank God he heard him screaming and pulled him out of the bin. Dad thought Derrick^a^ was at his grandmothers but the police called dad and told him to come to the police station and they said he had to sign Derrick^a^ over to care otherwise they would also take our baby too so dad had no choice but to sign him over to care. I was in the hospital 1000s miles away thinking my boy is going to die and dad calling me telling me about Derrick^a^ being taken away, I was broken, he is still in care but we will get him back when we get a place to live. It has affected us a lot because it has split us all up.”

^a^Not the child’s real name

### First nations health workers/ liaison officers

Most families expressed a lack of understanding about their child’s aftercare as well as a lack of understanding of the medical jargon used. Involvement of First Nations Health Workers and/or Liaison Officers working with health providers, the child and family members were voiced as important to improving communication, accessing ongoing healthcare and supporting the family (Table 8).

**Table 8:** Importance of First Nations health workers

Person-centred care and support	“We had to move from our community and no family is here but yeah, she [Aboriginal health worker] helped me with like food vouchers, and sit down and have a cup of coffee and a yarn at the hospital house they sent us to so Sonny^a^ could have dressings” “I ended up giving her a gift after she [Aboriginal Health Worker] done what she did, she supported me a lot and kept an eye on him and, so, I done her a nice painting she took home. She loved it.”
Facilitators to ongoing access to aftercare	“The [Aboriginal] health workers, they brought me up a few times for our appointments, I think a couple of times.” “We had to move into the hospital house and share with other Aboriginal families for a few months then they got us this house by ourselves. He needs to still see the physio. No, I don’t have a car, we either catch the bus to the hospital or the health girls [Aboriginal health workers] came and picked us up.” “Abby^a^ liked her [Aboriginal health worker] she was happy when she saw her the next time we went for dressing ‘cause we trusted her”
Communication	“It was so good having her [Aboriginal health worker] there when they were talking them big words she would tell us what they meant”

^a^Not the child’s real name

When families were asked to prioritise the importance of the themes emerging from this study, the families identified transport and dislocation from family and community, racism and lack of culturally safe communication as critical elements affecting burn aftercare. A significant positive facilitator to burn aftercare, mentioned by each family, was the importance of First Nations health worker who they described as having a role in maintaining the wellbeing of child, family and culture.

## Discussion

This is the first study that privileges the voices of Australia’s First Nations families on access to burn aftercare for children. Highlighted in this section are some of the significant systemic barriers to burn aftercare, which include but are not limited to, the lack of culturally appropriate communication, racism and distance from burn aftercare and family separation.

Issues around medical jargon and transport are also experienced by non-Indigenous Australians, however these themes that were identified by families are intensified from First Nations families. Whilst the issues regarding understanding of medical jargon affect non-Indigenous people from different racial backgrounds and those with limited literacy (particularly health literacy) this aspect of communication for Aboriginal and Torres Strait Islander peoples occurs on a background of ongoing colonisation and oppression as well as of intergenerational and individual trauma. Similarly, transport issues affect all rural and remote dwelling peoples, however, the context of colonisation means that the nature of these effects are experienced differently. Entrenched marginalisation in society has created contexts of poverty unique to First Nations peoples. Racism precludes First Nations peoples from fair access to public transport and private vehicle ownership. It is important to appreciate that some themes from this study may have broader applicability but there is a need to recognise and act upon the unique circumstances of First Nations families in order to improve experiences of ongoing care [21, 22]. The families that shared their experiences highlighted barriers which led to multiple and unnecessarily lengthy hospital visits for treatment and aftercare of their child’s burn injury.

### Racism

In line with the work of Durey, Thompson and Wood, who discussed the need to address institutional racism and misunderstandings in communication, our results similarly show that the issues are the same for burn aftercare and particularly emphasise the need for cultural change to address the systemic racism experienced by First Nations children and their families [23].

Racism is a word that continually comes to the forefront not only in Australia but globally and needs to be addressed if equity and equality are to be attained in healthcare systems. Western biomedical frameworks which reinforce colonial power structures, has been the platform on which health policies have been developed, and continues to marginalise Australia’s First Nations concept of health and wellbeing in our healthcare systems [24]. Institutionalised racism does not encourage ethical, respectful or culturally safe healthcare for Australia’s First Nations people; Durey and Thompson propose that *“In order to ensure culturally safe health service environments, both institutional and personal commitment is required”* [24].

Families spoke of a breakdown in communication within and from the multidisciplinary team which led to a lack of coordinated care between the tertiary and primary healthcare systems. Because there is no coordinated care between the systems, families missed appointments which further reinforced the stereotypical assumptions that First Nations families don’t attend follow-up appointments, when in fact they did not attend due to the breakdown in communication, false assumptions and racism.

Interpersonal and systemic racism by healthcare providers was experienced by the study families, discouraging them from attending future appointments. In addition, some children feared having to return for further treatment because of the culturally unsafe treatment they had previously received from the healthcare providers. The families reported that previous treatment by healthcare workers left their child and family members traumatised.

### Transport and family separation

A further barrier to children and families attending burn aftercare is the significant distance between burn aftercare facilities and family homes and the lack of transport services forcing dislocation and separation of families. This was demonstrated by Fraser et al. in a study which found that transport and distance from home and community were not taken into consideration in healthcare practices [5]. For instance, when one parent was caring for the child with the burn injury at the burns clinic, the other parent was elsewhere caring for the remaining children. Dislocation led to the family’s other children being at risk of removal by child services, this was highlighted by multiple families who participated in the yarning.

Families also spoke of a lack of planned processes for further management with the multidisciplinary team to ensure the best outcomes following their child’s discharge from the burns unit.

Some service providers encouraged relocation to the city from remote communities leading to displacement of the families. Families were told that their child would not receive the level of care needed, and that they should move from their community and home to live in the city.

Families shared how they felt these racist and colonialist statements did not take into consideration the importance of their connection to Country. To First Nations people, Country means connection, it is a spiritual belonging, and community and family are paramount to their psychosocial and cultural wellbeing. Families spoke about community being a place where many mothers and aunties help raise a child, and that comments like “you should move” amplify the issues of distance and led to experiences of hopelessness for First Nations families whose child required ongoing aftercare [25].

### First nations workers

Communication was expressed by the families as one of the leading barriers to accessing burn aftercare in Australia. An example in this study was use of medical jargon *‘... I didn’t understand what they were talking about, them words were too big medical words..’* to answer families’ questions which effectively left their questions unanswered.

However, the presence of First Nations Health Workers as mediators and interpreters of the medical jargon enabled the child and family to understand in lay terms what had happened and what would happen in the future regarding their child’s burn aftercare [26].

Parents acknowledged that without First Nations health worker involvement, there was no connection to culture for their child or family in the burn clinics. Furthermore, families stated that engagement of a First Nations Health Worker enabled trust between the service providers and child and/or family. Studies from Australia have shown that the involvement of First Nations Health Workers was crucial in creating culturally safe and appropriate healthcare systems for aftercare and cultivated trusting relationships between multidisciplinary team, the child and families [27].

The primary strength of this qualitative study is that it uses Indigenous methodologies and prioritises the voices of First Nations families whose child had sustained a significant burn injury and needed access to burn aftercare.

Another significant strength of this paper is in the design where following transcription, the transcripts were sent to families for confirmation of accuracy and feedback, reflecting an alignment of the families’ views and ensuring their voices represented the researcher’s interpretation of the journey of First Nations families whose child had experienced a burn injury.

A limitation of this study is that we were only able to recruit First Nations families whose child had experienced a serious burn injury from five hospitals in Australia where paediatric burn services were offered. First Nations children who were treated in outpatient centres not included here may have had different experiences that were not captured in this study.

## Conclusion

There is a lack of cultural competency and safety in healthcare for Australia’s First Nations people, creating barriers in access to health services, including burn aftercare. The lack of culturally competent communication causes fear, distrust and anxiety to the child and to family members. Aftercare cannot be separated from the initial care received, as it influences not only short-term but also long-term recovery for the child and the family.

A planned process enacted in a culturally appropriate and safe way would take into consideration the needs of all involved in the burn aftercare, including the multidisciplinary team, the First Nations Health Workers, the family and the child. Ensuring a clear pathway for the child’s aftercare needs, which is understood by the family, can lead to an easy transition to the multidisciplinary team from the burns unit to optimal healing for the child.

First Nations Health Workers had a positive influence on the impact for ongoing health and wellbeing as well as supporting the aftercare processes for healing. However, the role of First Nations Health Workers in supporting families and having an integral role in burn aftercare has not been recognised in Australia’s health systems to date [26, 28];. First Nations children who have experienced a serious burn and need a multidisciplinary team for ongoing aftercare to achieve a healthy, happy and productive life would benefit from the continued support and involvement of First Nations Health Workers.

The United Nations Declaration on the Rights of Indigenous Peoples clearly states that First Nations people globally have the right to best practice healthcare no matter where their choice of residence is [29]. Australia’s First Nations children also have the right to appropriate and beneficial aftercare for their burn injury to have the best outcomes for healing regardless of proximity to healthcare services.

*“Somewhere closer to [for bandages or physio] instead of travelling all that way. So you’re really far away from your family... I’d love it if they had it in [Country places] or something like that. Not saying that my kids are going to get burned again, but it’s just for all families.”* (Mother from remote community)

Collaborative and supported burn aftercare education and training for parents from experienced health workers to assist with home aftercare and added access to a burns unit for any queries parents may have will alleviate the need for constant long-distance travel, and therefore families can remain together and reduce dislocation.

The continued culturally unsafe practices experienced by the children and their families further reinforces racism in Australia. Health services in Australia need to incorporate culturally responsive healthcare as part of their routine burn aftercare. Training is necessary to foster good communication, stop racism and stereotyping, which in turn will encourage families to engage in aftercare appointments [30]. Training health workers in culturally responsive care ensures they are responsive to First Nations children and families’ needs in a vulnerable space.

## Data Availability

All data collected are stored in secure servers at The George Institute and to maintain Indigenous data sovereignty cannot be placed on an Open Data Platform.

## Abbreviations

HRECs: Human Research Ethics Committees
DoCS: Department of Community Services
OT: Occupational therapist
